# Decompressive craniectomy after unsuccessful intravenous thrombolysis of malignant cerebral infarction

**Published:** 2014-04-03

**Authors:** Humain Baharvahdat, Hamid Etemadrezaie, Samira Zabyhian, Zahra Valipour, Babak Ganjeifar, Seyed Mohammad Mousavi Mirzaye, Payam Sasannejad, Kavian Ghandehari

**Affiliations:** 1 Department of Neurosurgical, School of Medicine, Ghaem Hospital, Mashhad University of Medical Sciences, Mashhad, Iran; 2 Neurology Research Group, Student Research Committee, School of Medicine, Mashhad University of Medical Sciences, Mashhad, Iran; 3 Department of Neurosurgery, School of Medicine, Emdadi Hospital, Mashhad University of Medical Sciences, Mashhad, Iran; 4 Department of Neurology, School of Medicine, Ghaem Hospital, Mashhad University of Medical Sciences, Mashhad, Iran

**Keywords:** Cerebral Infarction, Decompressive Craniectomy, Ischemic Stroke, Thrombolysis

## Abstract

**Background:** Intravenous recombinant tissue plasminogen activator (rt-PA) is an approved treatment for acute ischemic stroke within 4.5 h of symptoms onset. Decompressive craniectomy (DC) has been shown as an effective therapeutic modality in malignant middle cerebral artery (MCA) infarction. As rt-PA could result in hemorrhagic complication during or after any surgery DC may be associated with severe bleeding after intravenous thrombolysis.

**Case Description:** A 57-year-old woman was presented 90 min after the sudden onset of left hemiplegia. Despite intravenous thrombolytic therapy, she lost consciousness within 48 h and brain CT scan showed a right malignant MCA infarction associated with a small bleeding. DC was performed without any complication. The patient improved dramatically.

**Conclusion:** DC could be done safety for malignant MCA infarction after unsuccessful intravenous thrombolytic therapy even the later was complicated with intra-infarction hemorrhage.

## Introduction

Intravenous thrombolysis is approved for acute ischemic stroke within 4.5 h of onset in our hospital. Decompressive craniectomy (DC) was shown to be lifesaving and effective for malignant middle cerebral artery (MCA) infarction.^1^^-^^4^ There is a risk of bleeding when DC performed after thrombolytic treatment of acute ischemic stroke. Herein, we present a case of DC after unsuccessful intravenous recombinant tissue plasminogen activator (rt-PA) treatment of MCA infarction.

## Case Reports

**Figure 1:**
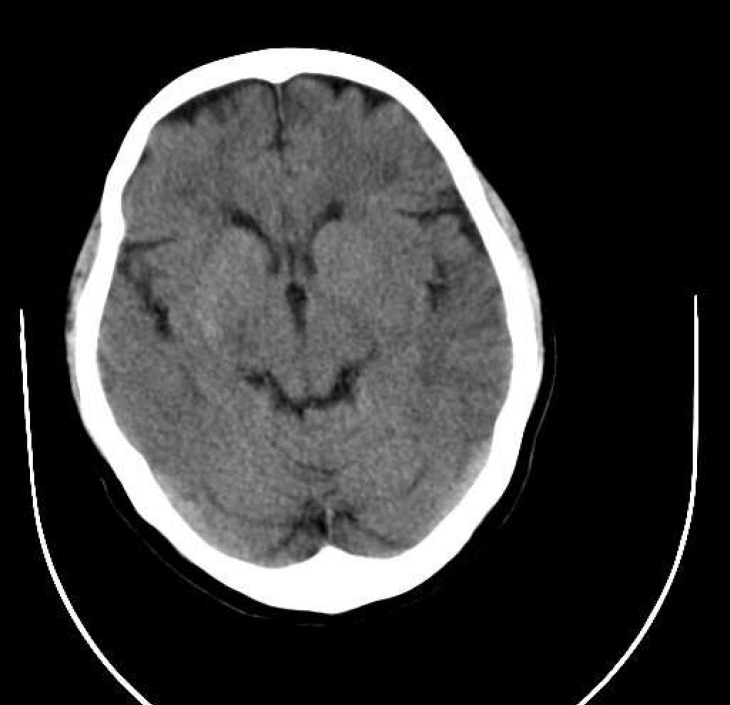
CT scan prior to tissue plasminogen activator administration

**Figure 2:**
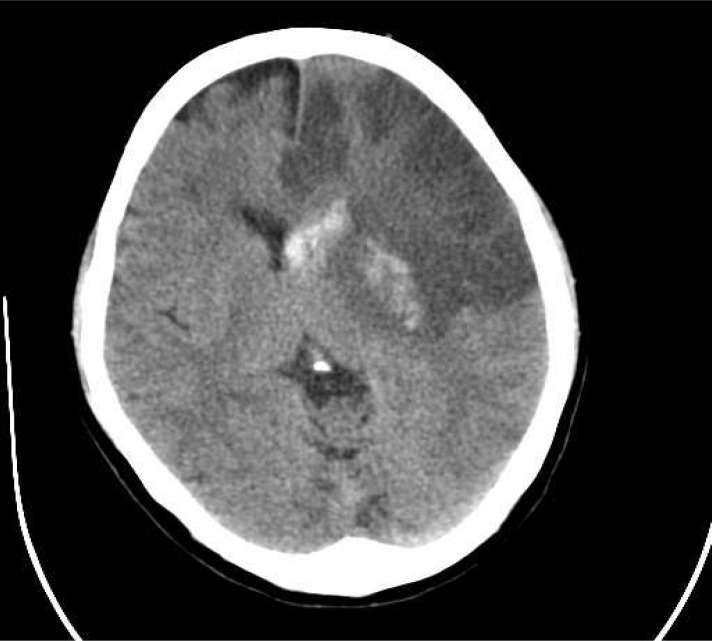
CT scan 48 h after tissue plasminogen activator administration

**Figure 3:**
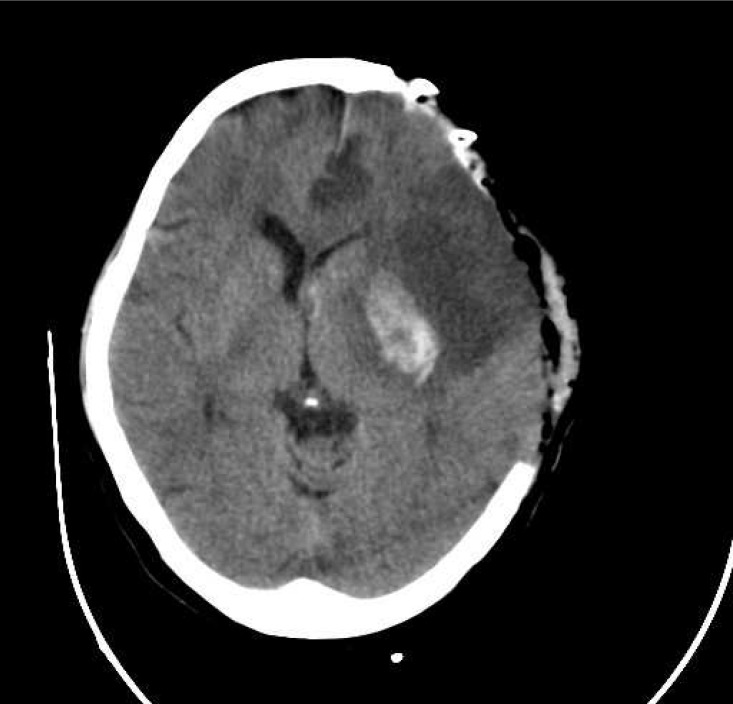
CT scan after decompressive craniectomy

The case we present here is about a 57-year-old woman who was presented with sudden onset of right hemiplegia. On physical examination, she was alert with right hemiplegia, right facial paresis, and severe aphasia (National Institute of Health Stroke Scale [NIHSS], 15 points). She had untreated mitral valve stenosis. The time between the onset of symptoms and admission was 90 min. Emergent brain CT scan showed effacement of left MCA territory (Figure 1). Thrombolytic therapy with standard dose of intravenous rt-PA (0.9 mg/kg) was performed. During the next 48 h, her health deteriorated, and her level of consciousness decreased (NIHSS 18). Brain CT scan results were consistent with an extensive infarction of left MCA territory with hemorrhage in basal ganglia region and midline shift (Figure 2). After that, a large Decompressive hemicraniectomy, >12 cm in anterio-posterior diameter and >8 cm in inferio-superior diameter, was performed with duroplasty (Figure 3). The bone flap was preserved in the abdominal wall. The following day, brain CT scan revealed resolution of midline shift without any new hematoma. She improved progressively during the following day, and on the 15^th ^day of operation she was discharged with NIHSS 11. Three months later, she was admitted for cranioplasty. She was alert with NIHSS 4. The cranioplasty was performed without any complication.

## Discussion

Recently, pooled data from three European randomized controlled trials (DECIMAL, DESTINY, and HAMLET trials) have shown that not only DC has dramatically decreased the mortality rate of malignant MCA infarction from 80% to 20%, but also improved patients’ functional outcome.^1^^-^^4^

Recombinant t-PA enhances local fibrinolysis by converting plasminogen to plasmin. Its half-life is <5 min. Its clearance rate is 380–570 mL/min and is dominantly through the liver.^5^^,^^6^ Despite short half-life of rt-PA (5 min), its fibrinolytic effect may persist up to 24–48 h.^7^ Symptomatic intra-cerebral hemorrhage is the most severe complication of thrombolytic therapy with rt-PA that complicates 6.4% of patients within 36 h of treatment.^8^ Therefore, there is a major concern about DC after thrombolysis because it may be complicated by severe hemorrhage during or after surgery or result in progression of the preexisting intracranial hematoma. Williams et al. represented two cases of malignant MCA infarction, managed by DC after 24 h of intravenous rt-PA administration. The procedures were reported without complication, and both patients improved.^9^ Fisher et al. showed that the mortality and major complications after DC in malignant MCA infarction were not different between patients with prior intra-arterial thrombolysis (IAT) and those without prior IAT.^10^ One of their patients with prior IAT was complicated with severe hemorrhage during DC probably due to pretreatment with dual anticoagulant therapy of aspirin and plavix rather than to IAT alone.^10^ Takeuchi et al. also revealed that patients with malignant MCA infarction there was no difference for new intracranial bleeding and worsening of pre-existing intra-cerebral hemorrhage between the DC patients with prior intravenous thrombolysis (IVT) and those without prior IVT.^11^ In both studies, DC resulted in a favorable outcome in patients with prior IVT or IAT similar to HAMLET study.^10^^,^^11^ Our patient also profited DC without any hemorrhagic complication despite prior IVT. These results may suggest the safety and efficacy of DC after IVT or IAT for malignant MCA infarction.

The optimal time of DC after thrombolysis remains to define. Although the fibrinolytic effect of rt-PA could persist up to 24-48 h,^7^ previous studies have shown that DC even within first 48 h of rt-PA administration could be performed safely.^9^^-^^11^ Fibrinogen degradation products (FDP), α2-plasmin inhibitor, and the plasmin-α2-plasmin inhibitor change dramatically during 24 h and they retain to their baseline level within 24 h.^7^ It is recommended to perform DC within 48 h of malignant MCA infarction occurrence.^4^^,^^12^ It is assumed that the optimal time for DC could be between 24 and 48 h after rt-PA administration. In addition, the serum levels of FDP, α2-plasmin inhibitor, and the plasmin-α2-plasmin inhibitor before DC could be helpful for precise decision of optimal time of DC.
